# Evaluating PRN Medication Prescribing Practices in Mental Health Services: A Comparative Audit Following a Serious Incident

**DOI:** 10.1192/bjo.2025.10672

**Published:** 2025-06-20

**Authors:** Nidhi Shashidhar, Olayinka Adegboye

**Affiliations:** Nottinghamshire Healthcare NHS Foundation Trust, Nottinghamshire, United Kingdom

## Abstract

**Aims:** PRN (pro re nata) medications are widely used in mental health settings but are prone to misuse and prescribing errors. A serious incident involving a patient’s death linked to excessive PRN medication supply prompted an initial audit to evaluate compliance with prescribing standards. A re-audit was conducted to assess progress and identify ongoing challenges.

**Methods:** Two prospective audits were conducted across an inpatient acute ward and a rehabilitation centre. The initial audit (29/07/2024–06/08/2024) and re-audit (29/01/2025–06/02/2025) reviewed medication cards, Rio (electronic patient notes) and EPMA (Electronic Prescribing and Medicines Administration) for 31 patients prescribed PRN medications. Compliance was assessed against 13 predefined standards, including generic naming, dose intervals, BNF compliance, and regular reviews.

**Results:** Sustained Full Compliance:

Both audits demonstrated 100% compliance in key areas: generic naming, specified administration routes, separate prescriptions for multiple routes, adherence to BNF limits, clear indications for use, and rewriting altered prescriptions.

Key Improvements:

Minimum dose interval specification improved from 64.5% to 93.5%.

Maximum dose documentation increased from 96.7% to 100%.

Regular ward round reviews rose dramatically from 3.2% to 64.5%.

Discontinuation of unused PRN medications (>1 month) improved from 0% to 22.2%.

Review of PRN medications used regularly (>72 hours) increased from 0% to 28.5%.

Documentation of regular vs. PRN use improved from 33.3% to 44.4%.

Ongoing challenges:

Review of PRN medications used regularly (>72 hours) remained low at 28.5%.

Discontinuation of unused PRN medications (>1 month) was only 22.2%.

Documentation of regular vs. PRN use remained below 50%.

**Conclusion:** The re-audit demonstrates significant progress in dose interval specification, maximum dose documentation, and ward round reviews. However, challenges persist in the regular review and discontinuation of PRN medications, as well as in documenting regular vs. PRN use. Continued focus on these areas is essential to ensure patient safety and adherence to best prescribing practices.

Recommendations:

Key recommendations include integrating PRN standards into doctor inductions, involving pharmacists in ward rounds, and conducting regular re-audits to monitor progress and sustain improvements. Disseminating guidelines and providing feedback to medical teams are essential steps toward achieving full compliance and enhancing patient safety.

